# Formulation and *in vitro* Evaluation of Floating Capsules of Theophylline

**DOI:** 10.4103/0250-474X.41460

**Published:** 2008

**Authors:** S. B. Bhise, N. H. Aloorkar

**Affiliations:** * Government College of Pharmacy, Karad-415 124, India; 1 Department of Pharmaceutics, Satara College of Pharmacy, Satara-415 004, India

**Keywords:** Theophylline, floating drug delivery system, sustained release

## Abstract

Sustained release floating capsules for theophylline were fabricated using drug:polymer ratio of 30:70. The hydrocolloids were used in different proportions and four formulations were prepared. These formulations were optimized on the basis of buoyancy, matrix integrity, duration of floating and *in vitro* drug release. All the four formulations showed good buoyancy and matrix integrity. The duration of floating was more than 12 h for all formulations. *In vitro* drug release study of these formulations indicated controlled release of theophylline and about 76 percent drug was released at the end of 12 h.

Floating drug delivery system is an oral dosage form designed to prolong the residence time of dosage form within the GI tract^1^. Such dosage form having density less than that of the gastric fluid floats on the gastric juice for an extended period of time while slowly releasing the drug. On contact with the gastric fluid, the intragastric floating capsule forms a water impermeable colloid gel barrier around its surface and maintains a bulk density of less than 1. So, it remains buoyant in the gastric fluid in stomach until the entire loading dose has been released. This drug delivery system not only prolongs GI residence time but does so in an area of the GI tract that could maximize drug reaching its absorption site in solution and hence ready for absorption. In the present study, hydrodynamically balanced system approach has been selected to control the delivery of theophylline for longer period in stomach from floating drug delivery system.

Theophylline, one of the most popular drugs, is used to treat bronchial asthma. Peak serum theophylline concentration occurs 1-2 h after ingestion of liquid preparations, capsules and uncoated tablets and between 4 and 12 h after ingestion of sustained release preparations. Optimum serum theophylline concentration ranges from 10-20 μg /ml^2^–^6^. It has an average half-life of 8.7 h but in case of smokers, it is 4.4 h. Hence, it requires frequent dosing for achieving therapeutic drug concentration in the target tissue. The objective of present study was to formulate floating capsules of theophylline to deliver the drug continuously with set limits of dissolution profile and minimum floating time of 8 h.

Theophylline was obtained from Nucron Pharmaceuticals Ltd, Pune. Hydroxypropylmethylcellulose (HPMC K4M) and hydroxyethylcellulose (HEC) was obtained from Amrut Pharmaceuticals, Thane. Ethylcellulose (EC), methylcellulose (MC K4 M) and sodium carboxymethylcellulose (Na-CMC) was obtained from Loba Chemie, Mumbai. The other excipients used were calcium phosphate dibasic, mannitol, and glycerol monostearate and magnesium stearate. They were of analytical grade and were used as procured.

Before actual formulation, an initial study was carried out to find out the optimum combination of drug and polymers. For floating capsules, hydrocolloids of natural as well as semi synthetic origin were selected. The hydrocolloids selected were hydroxypropylmethylcellulose, hydroxyethylcellulose, methylcellulose and sodium carboxymethylcellulose. In addition to these hydrocolloids, tragacanth was also used. The drug and polymers were taken in a ratio ranging from 90:10 to 10:90. This was done to select the optimum combination of drug polymer ratio in the floating drug delivery device in such a way that it would pass the tests of buoyancy, matrix integrity and duration of floating in 0.1 N HCl. The other excipients used were calcium phosphate dibasic, ethyl cellulose, mannitol, and glycerol monostearate and magnesium stearate. The hydrocolloids alongwith the excipients were blended homogenously with the drug. The blended mixture was then filled in the colorless transparent gelatin capsules.

The filled capsules were then observed for buoyancy, matrix integrity and duration of floating (Table 1). From the Table, it was clear that formulation G containing drug and polymers in the ratio of 30:70 remained buoyant in 0.1 N HCl for more than 12 h and maintained the shape. So this combination was selected for further study to incorporate the dose of 100 mg of theophylline. After selecting the ideal combination (30:70, drug: polymer), the actual formulations were prepared. The dose of theophylline was taken to be 100 mg. Taking 100 mg as the 30 percent, the quantity of polymers was calculated which came out to be 233.3 mg. Four different formulations were prepared using the polymers in different proportions keeping the concentrations of other excipients constant (Table 2).

**TABLE 1:** DRUG: POLYMER COMBINATION

Formulation	Buoyancy	Matrix integrity	Duration of floating (h)
A (90:10)	-	-	-
B (80:20)	+	-	2
C (70:30)	+	-	2.3
D (60:40)	+	-	6
E (50:50)	+	+	6.4
F (40:60)	+	+	7
G (30:70)	+	+	>12
H (20:80)	+	+	>12
I (10:90)	+	+	>12

-denotes non-buoyant/non-intact capsule and + indicates buoyant/intact capsule

**TABLE 2:** FORMULATIONS OF FLOATING CAPSULES OF THEOPHYLLINE

Ingredient	Formulae of theophylline floating capsules Qty (mg)			
	
	1	2	3	4
Theophylline	100	100	100	100
HPMC K4 M	93.32	69.99	46.66	23.33
MC K4 M	69.99	46.66	23.33	93.32
HEC	46.66	23.33	93.32	69.99
Na-CMC	23.33	93.32	69.99	46.66
Glycerol monostearate	6	6	6	6
Calcium phosphate dibasic	20	20	20	20
Mannitol	14	14	14	14
Ethyl cellulose	15	15	15	15
Magnesium stearate	13	13	13	13
Tragacanth	10	10	10	10

Formulation 1: HPMC:MC:HEC:Na-CMC.2:1.5:1:0.5; Formulation 2: HPMC:MC:HEC:Na-CMC-1.5:1:0.5:2; Formulation 3: HPMC:MC:HEC:Na-CMC-1:0.5:2:1.5 and Formulation 4: HPMC: MC:HEC: Na-CMC - 0.5:2:1.5:1

All the ingredients in the above formulations were weighed accurately and ground individually. All these ingredients were mixed with the drug homogenously and filled in the colorless transparent gelatin capsules. Batch size for all formulations was 100 capsules. The capsules of these formulations were evaluated for buoyancy, matrix integrity, duration of floating, weight variation and *in vitro* drug release characteristics. Floating time was determined using U.S.P.24 dissolution apparatus2^7^ at 100 rpm using 900 ml of 0.1 N HCl and temperature was maintained at 37±0.5^°^ throughout the study. The duration of floating is the time the capsule floats in the dissolution medium.

*In vitro* drug release was studied using USP 24 paddle dissolution apparatus II^7^ in 900 ml of 0.1 N HCl at 37±0.5^°^ and at 100 rpm. An aliquot of 2 ml of the sample was withdrawn at regular intervals and the same volume of prewarmed (37±0.5^°^) fresh dissolution medium (0.1 N HCl) was replaced. The samples withdrawn were filtered and drug content in each sample was analyzed after suitable dilution by GBC-UV/Vis 911A Spectrophotometer at 270 nm. The actual drug content in the formulations was then calculated from the standard curve prepared with theophylline in 0.1 N HCl.

Capsules of all the formulations were found to pass the tests for physical parameters like buoyancy, matrix integrity and duration of floating (Table 3). All the capsules were found to be floating for more than 12 h. The drug content in the capsules of each formulation was found within the range of 90-110 percent as specified for theophylline capsules IP^8^, which confirms to show an excellent drug content uniformity in each formulation (Table 3).

**TABLE 3:** TABLE SHOWING BUOYANCY, MATRIX INTEGRITY AND DURATION OF FLOATING

Formulation	Buoyancy	Matrix integrity	Floating duration (h)	Drug content uniformity	Average weight (mg)±mean % deviation
1	+	+	>12	99.20	395.6±0.05
2	+	+	>12	98.6	395±0.03
3	+	+	>12	97.36	393.5±0.7
4	+	+	>12	98.25	396±0.9

± Buoyant/ Intact capsules

All the formulations confirmed to the general pharmacopoeial requirement of not more than ±7.5 % deviation^9^. Capsules of all formulations showed an excellent uniformity in their weights. *In vitro* drug release studies from formulation 1-4 showed the release of 76.54%, 75.10%, 66.04% and 70.05% which clearly confirm that formulation 1 containing HPMC: MC:HEC: Na-CMC in the proportion of 2:1.5:1:0.5 exhibited better release from the capsules ((fig. 1), Table 4)

**TABLE 4:** *IN VITRO* DRUG RELEASE DATA OF FLOATING CAPSULES OF THEOPHYLLINE

Formulation	% cumulative drug release			
	
	3 h ±SD	6 h ±SD	9 h ±SD	12 h±SD
1	35.95±1.436	55.62±1.318	67.53±1.141	76.54±1.792
2	41.58±1.146	54.40±1.414	66.79±0.750	75.10±2.005
3	32.64±2.285	48.28±2.627	62.16±1.258	66.04±2.118
4	33.64±0.754	50.46±1.171	62.66±1.722	70.05±1.337

Mean of Triplicate studies. Each capsule contains theophylline 100 mg

**Fig. 1:**
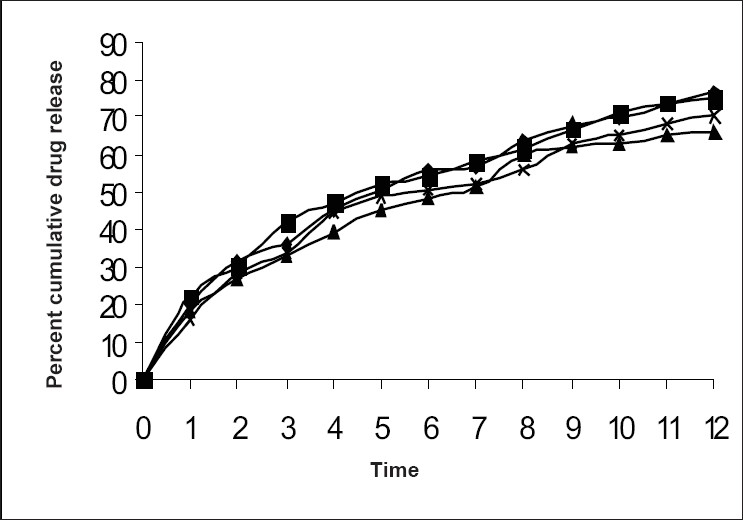
*In vitro* dissolution profile of theophylline from formulations 1 to 4 in 0.1 N HCl.
